# Membrane-Associated Transporter Protein (MATP) Regulates Melanosomal pH and Influences Tyrosinase Activity

**DOI:** 10.1371/journal.pone.0129273

**Published:** 2015-06-09

**Authors:** Bum-Ho Bin, Jinhyuk Bhin, Seung Ha Yang, Misun Shin, Yeon-Ju Nam, Dong-Hwa Choi, Dong Wook Shin, Ai-Young Lee, Daehee Hwang, Eun-Gyung Cho, Tae Ryong Lee

**Affiliations:** 1 Bioscience Research Institute, Amorepacific Corporation R&D Center, Yongin, Republic of Korea; 2 Department of Chemical Engineering, POSTECH, Pohang, Republic of Korea; 3 Gyeonggi Bio Center, Gyeonggi Institute of Science & Technology Promotion, Suwon, Republic of Korea; 4 Department of Dermatology, Dongguk University Ilsan Hospital, Goyang, Republic of Korea; University of Alabama at Birmingham, UNITED STATES

## Abstract

The *SLC45A2* gene encodes a Membrane-Associated Transporter Protein (MATP). Mutations of this gene cause oculocutaneous albinism type 4 (OCA4). However, the molecular mechanism of its action in melanogenesis has not been elucidated. Here, we discuss the role of MATP in melanin production. The *SLC45A2* gene is highly enriched in human melanocytes and melanoma cell lines, and its protein, MATP, is located in melanosomes. The knockdown of MATP using siRNAs reduced melanin content and tyrosinase activity without any morphological change in melanosomes or the expression of melanogenesis-related proteins. Interestingly, the knockdown of MATP significantly lowered the melanosomal pH, as verified through DAMP analysis, suggesting that MATP regulates melanosomal pH and therefore affects tyrosinase activity. Finally, we found that the reduction of tyrosinase activity associated with the knockdown of MATP was readily recovered by copper treatment in the *in vitro* L-DOPA oxidase activity assay of tyrosinase. Considering that copper is an important element for tyrosinase activity and that its binding to tyrosinase depends on melanosomal pH, MATP may play an important role in regulating tyrosinase activity via controlling melanosomal pH.

## Introduction

Melanin production in humans is an essential cellular response in the eyes, hair and skin to protect the cells from harmful ultraviolet light, reducing the risk of melanoma progression [1–4]. Melanin is commonly produced by melanocytes that originated from the neural crest and reside in the basal layer of the epidermis [5]. Skin color determinant melanin is divided into two groups, eumelanin and pheomelanin, and functions to protect the DNA from damage by absorbing ultraviolet light [6, 7]. Eumelanin is a black or brown color and is responsible for black or brown human skin and hair. Pheomelanin is a reddish color and is responsible for red hair [6, 8].

The lack of or reduction in melanin production in skin and hair is called albinism [9]. Melanin deficiencies due to poor melanin production in the eyes, hair and skin, associated with impaired vision and skin that is easily damaged by sunlight, is called oculocutaneous albinism (OCA) [9]. This condition commonly results from alterations in melanogenesis-related proteins or mutations in tyrosinase (OCA1), pink-eyed dilution protein (OCA2), tyrosinase-related protein 1 (OCA3) and membrane-associated transporter protein (OCA4) [10, 11]. There are severe forms of albinism involving pathological alterations, such as Hermansky-Pudlak syndrome (HPS) and Chediak-Higashi syndrome (CHS) [12]. HPS is an autosomal recessive disorder caused by mutations in HPS family members involving bleeding and cellular storage disorders [12]. The *HPS* gene encodes a protein that plays crucial roles in organelle biogenesis in normal cells, which is also important in melanosome production. The loss of its function following a mutation causes albinism [12]. In a similar fashion, the *CHS1* gene participates in the regulation of lysosomal trafficking in normal cells, including melanocytes. The loss of its function by a mutation leads to Chediak-Higashi syndrome and albinism [13, 14].

The *OCA4* gene encodes a sugar transporter-like membrane protein known as the Membrane-Associated Transporter Protein (MATP) [15, 16]. MATP, encoded by *AIM1* in medaka [17], is also known as SLC45A2 and is a member of the solute carrier family 45A. The SLC45 family consists of four members, SLC45A1, SLC45A2 (MATP), SLC45A3, and SLC45A4 [18], and has high similarity to a recently identified functional animal sucrose transporter (SCRT) in *Drosophila melanogaster* [19]. SCRT is similar to plant sugar uptake transporters (SUTs) containing a typical sucrose transporter sequence (RxGRR) [19]. Interestingly, the expression of SCRT is enriched in melanin-containing organelles as well as in the gastrointestinal tract, suggesting a possible role for sucrose transporters in melanin synthesis [19]. In the SLC45A2 gene, a T-to-C transition in codon 435 and a G-to-A transition in codon 153, which result in S435P and D153N mutations, respectively, are related to OCA4, a hypopigmentation disorder with alterations in skin/hair/eye pigmentation [20]. In addition, a pigmentation variation caused by the single nucleotide polymorphisms F374L and E272K in MATP has been reported [21]. In a study of primary melanocytes isolated from mice deficient in the underwhite (*uw*) gene, a homologue of human MATP, correct tyrosinase processing but not intracellular trafficking to the melanosomes was observed [16]. Although the functional relevance of MATP in melanogenesis is strongly suggested via the phenotypic analysis of humans harboring mutations in the SLC45A2 gene or mice deficient in the *uw* gene, a molecular mechanism to explain how MATP regulates melanin biosynthesis in humans has not been elucidated.

In this study, we verified the role of MATP in melanogenesis using human primary melanocytes and melanoma cells. Among SLC45A family genes, only SLC45A2 was enriched in the tested human melanocytes and melanoma cells and its protein, MATP, was located in melanosomes. The knockdown of MATP using siRNA reduced melanin content and tyrosinase activity by acidifying the pH of the melanosomes. Finally, we proposed a model explaining how MATP regulates melanogenesis.

## Results

### The SLC45A2 transcript is highly expressed in human melanoma cells and primary melanocytes

**Fig 1:**
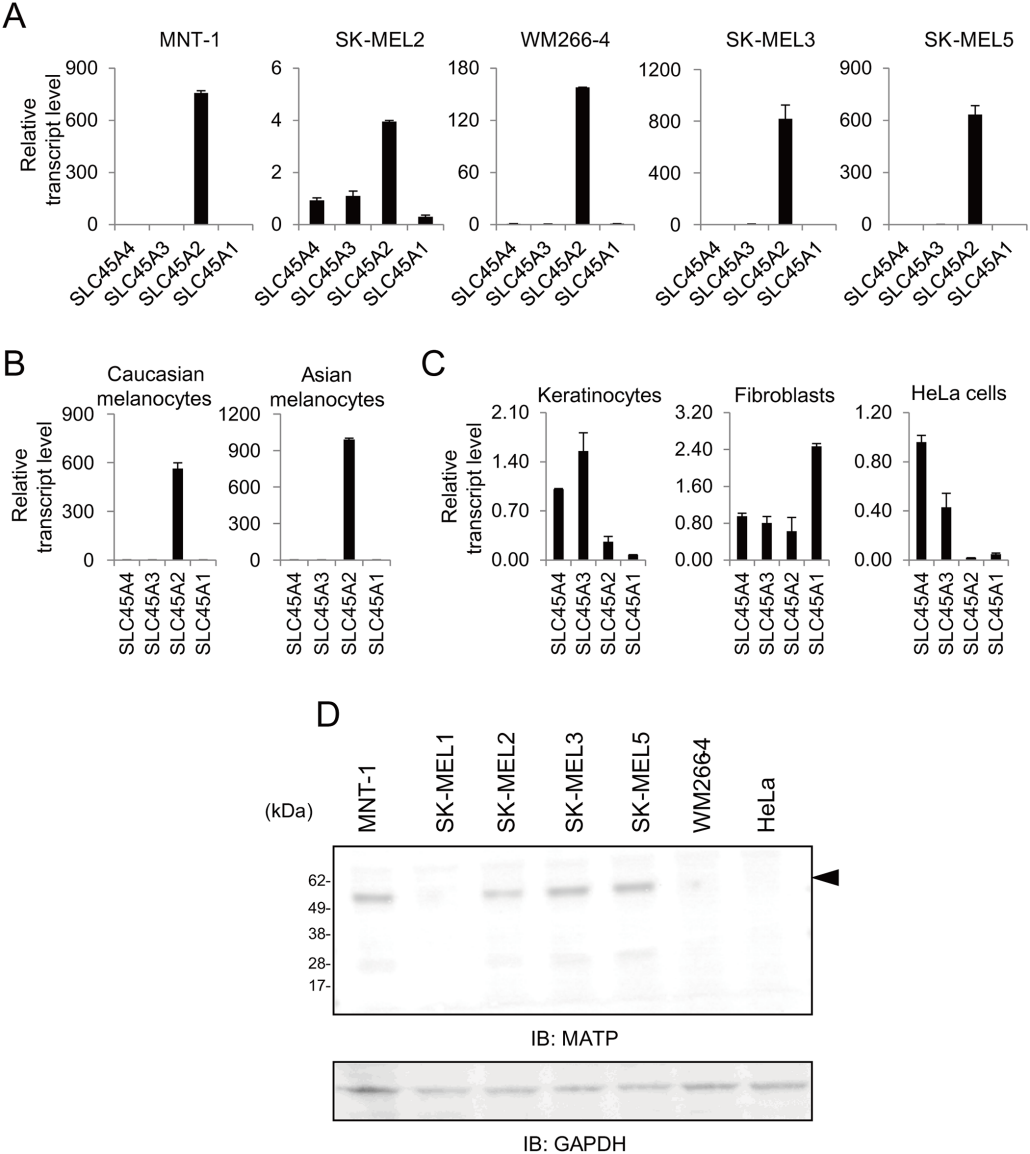
The SLC45A2 transcript is highly expressed in human melanoma cells and primary melanocytes. Expression of the SLC45A transcripts in various human melanoma cell lines (A), normal melanocytes from Caucasians and Asians (B), and human non-melanogenic skin cells (C) were examined by RT-qPCR. The data are representative of three independent experiments. (D) The expression of the MATP protein in human cells was analyzed by immunoblotting with an anti-MATP antibody. The arrowhead indicates the MATP protein. The data are representative of three independent experiments.

At least 4 transcripts of the SLC45A family have been described in the human genome [18]. Despite their high functional and structural similarity, SLC45A family members, other than SLC45A2, have not been studied in relation to pigmentation. We determined the expression pattern of the SLC45A family members in various pigmented cells, human melanoma cell lines and primary melanocytes and in several non-pigmented cells using quantitative real-time polymerase chain reaction (qRT-PCR). Among the SLC45A members, the SLC45A2 transcript was highly enriched in the majority of the melanoma cell lines (Fig 1A). The expression pattern of SLC45A2 was determined in normal melanocytes derived from Caucasians and Asians (Fig 1B) but not in non-melanogenic cells, keratinocytes, fibroblasts, or HeLa cells (Fig 1C). Compared to SLC45A2, the expression of SLC45A3 and SLC45A4 was highly enriched in keratinocytes and HeLa cells and SLC45A1 in fibroblasts (Fig 1C). The expression of MATP protein, which is encoded by the *SLC45A2* gene, was analyzed by western blotting with anti-MATP antibodies. A major band detected by three different antibodies was approximately 58 kDa in molecular weight, which is very close to the size of MATP protein (approximately 58.3 kDa) calculated from amino acid sequence, and this band was not detected in HeLa cells in all analyses (Fig 1D and S1 Fig). These results indicated that among the SLC45A family members, SLC45A2 is specifically expressed in human melanocytic lineages, such as melanoma cell lines and melanocytes, and could play a specific role(s) in melanogenesis.

### The MATP protein is located in melanosomes

**Fig 2:**
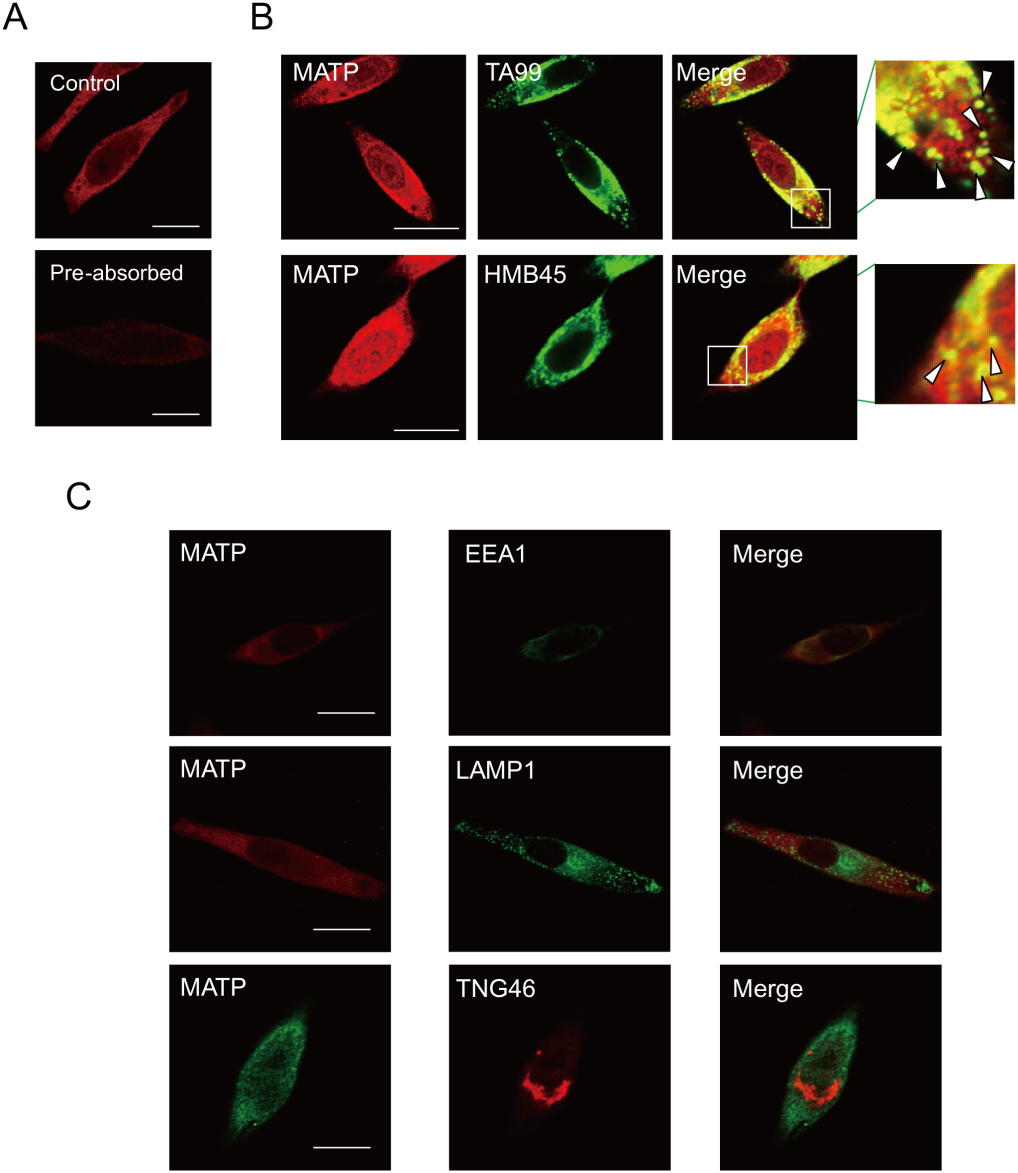
The MATP protein is located in the melanosomes. (A) MNT-1 cells were stained with anti-MATP antibody before (MATP) or after antigen pre-absorbance (Pre-absorbed) for 5 min prior to adding an anti-MATP antibody. (B) MNT-1 cells were stained with anti-TA99 or-HMB45 (a melanosomal protein) antibodies together with an anti-MATP antibody. The insets are magnified, and the co-localization of MATP and each melanosomal marker is indicated by arrowheads. Scale bars = 10 μm. (C) Cells were stained with anti-EEA1, anti-LAMP1, or anti-TNG46 antibodies to detect the endosomes, the lysosomes, or the Golgi apparatus, respectively, together with an anti-MATP antibody. These representative images were captured by confocal microscopy. Scale bars = 10 μm.

MATP is associated with OCA4 and has been proposed as a melanosomal protein [22]. The LC/MS data implied that the MATP protein is located in the melanosomes [23]. Using an anti-MATP antibody, which detected the MATP protein as a single major band in western blot analysis (Fig 1D, arrowhead) and reduced the signal from MATP after being pre-absorbed by MATP antigen (Fig 2A), we performed confocal microscopic analysis to observe the cellular location of the endogenous MATP protein. Its expression was partially merged with the melanosomal markers TA99 and HMB45 (Fig 2B and S2 Fig, insets, arrow heads) and with EEA1, an endosomal marker (Fig 2C, first panel), but not with LAMP1, a lysosomal marker (Fig 2C, second panel), or TNG46, a Golgi apparatus marker (Fig 2C, third panel). These data indicate that the MATP protein was specifically present in both endosomes and melanosomes but not in the Golgi apparatus or the lysosomes, implying its specific involvement in melanogenesis.

### The knockdown of MATP using siRNA (MATP-KD) reduces melanin production

**Fig 3:**
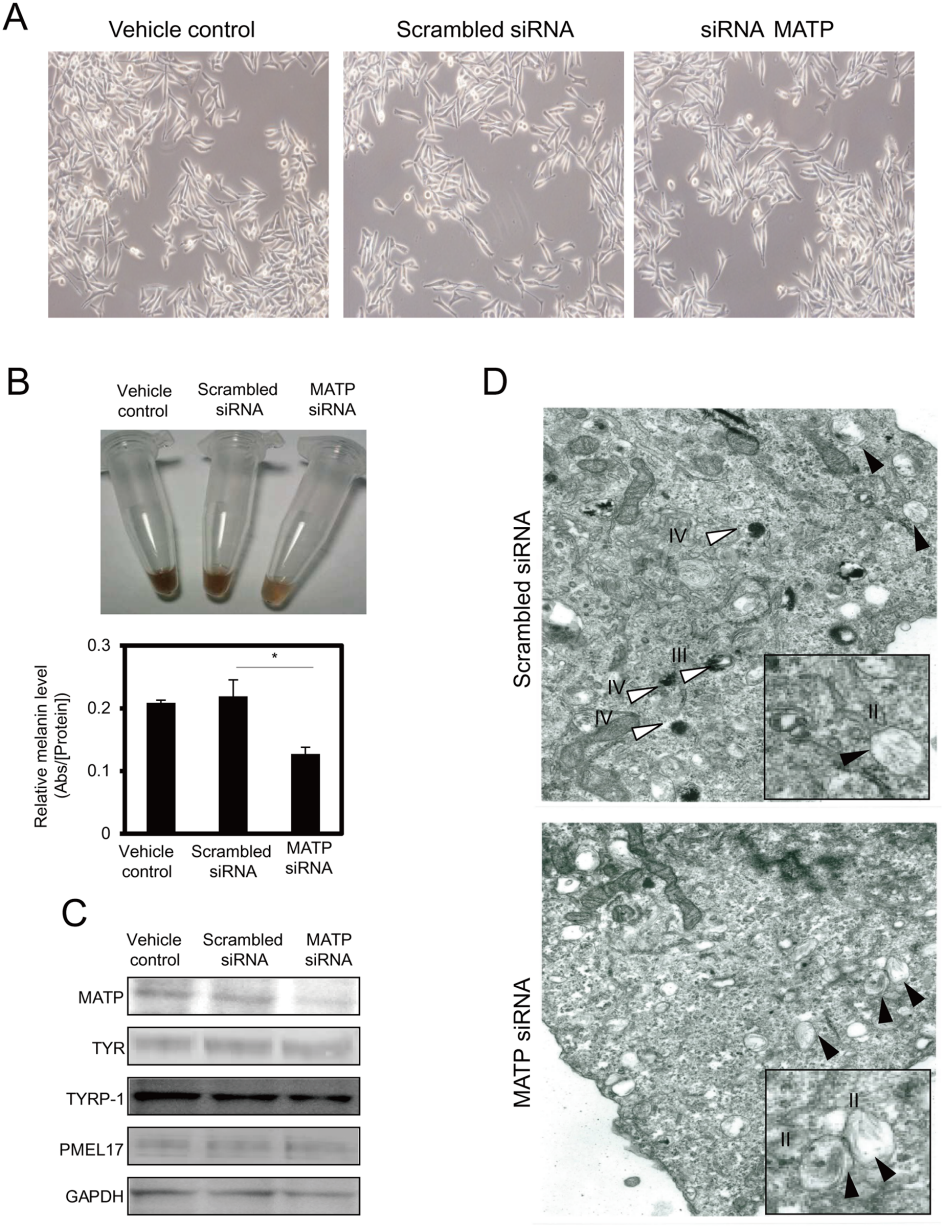
The knockdown of MATP using siRNA reduces melanin production. (A) The representative microscopic images after each siRNA treatment for 4 days. (B) The color of the cell lysates from 2 x 10^5^ MNT-1 cells was monitored after each treatment, and the melanin contents were measured by measuring the absorbance at 450 nm. The data are representative of three independent experiments (*, *P* < 0.05). (C) The expression level of melanogenesis-related proteins in MNT-1 cells was analyzed by immunoblotting with the appropriate antibodies. TYR, tyrosinase; TYRP-1, tyrosinase related protein-1; PMEL17, premelanosome protein 17. (D) Electron microscopy analysis. The white and black arrowheads indicate mature (stage III or IV) and early (stage II) melanosomes, respectively. II, stage II melanosome; III, stage III melanosome; IV, stage IV melanosome.

**Fig 4:**
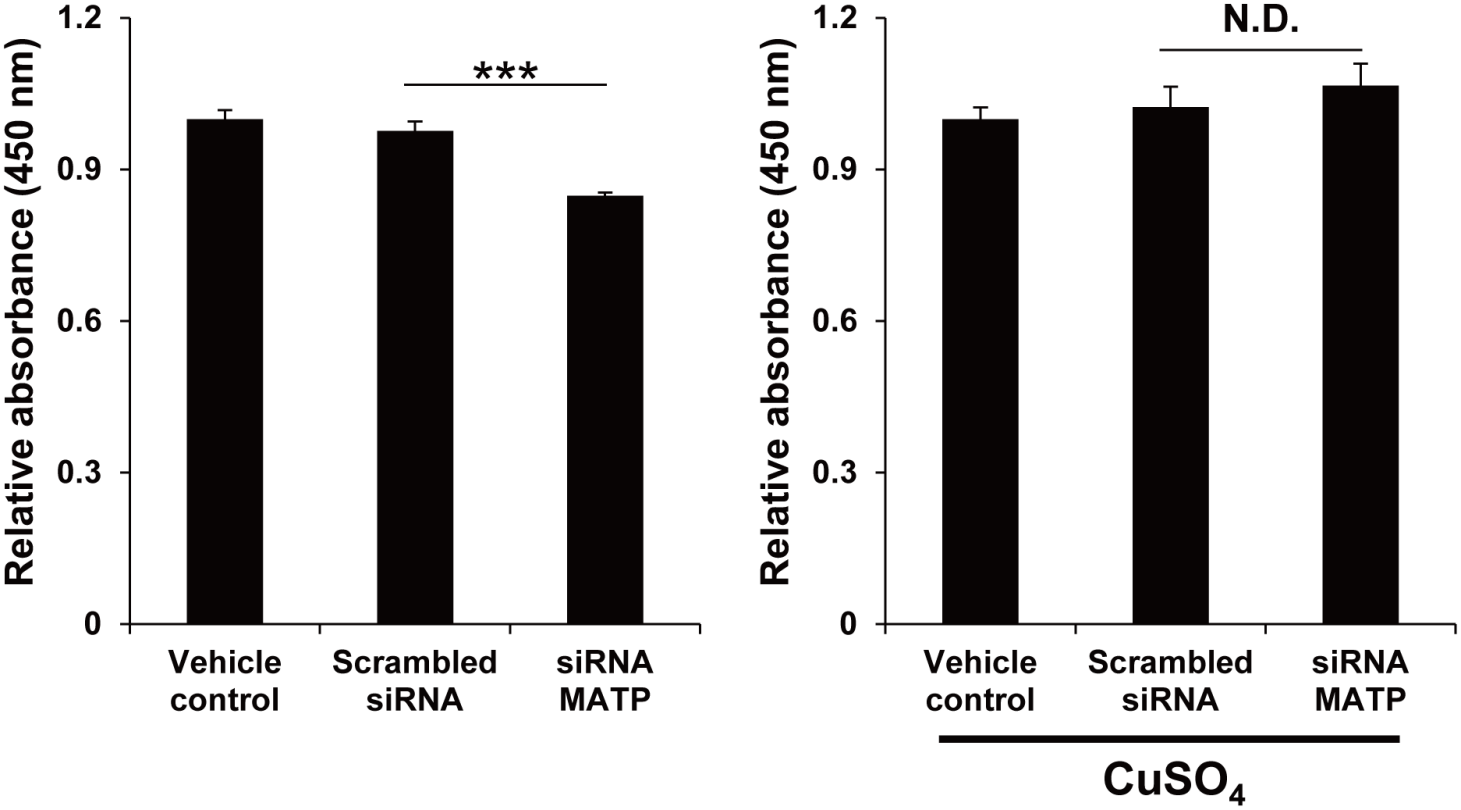
Copper treatment recovers the L-DOPA oxidase activity of tyrosinase in MATP-KD cells. MNT-1 cells were treated with each siRNA for 2 days. The lysates were treated with or without 1 mM CuSO_4_ for 5 min before incubating with 2 mg/ml L-DOPA for 1 hour, after which the absorbance at 450 nm was measured. The data are representative of three independent experiments (***, *P* < 0.005).

To examine the effect of the MATP protein in melanogenesis, we knocked down endogenous MATP using siRNA in MNT-1 cells. MATP-KD did not alter cellular morphology (Fig 3A) but reduced the melanin content (Fig 3B) and the L-DOPA oxidase activity of tyrosinase (Fig 4). We examined the expression of the melanogenesis-related genes and proteins after MATP deletion. Treatment with MATP siRNA reduced the mRNA and protein levels of endogenous MATP compared to vehicle or scrambled siRNA-treated controls (Fig 3C and S3 Fig). Interestingly, the mRNA and protein levels of melanogenesis-related genes, such as tyrosinase, tyrosinase related protein 1 (TYRP-1), premelanosome protein 17 (PMEL17) and microphthalmia-associated transcription factor (MITF), were not altered by MATP-KD (Fig 3C and S3 Fig). We also analyzed whether there was any change in N-glycan modification in tyrosinase because N-glycosylation is an important factor for tyrosinase activity, which was reduced in the MATP-KD cells (Fig 4). We did not observe any difference in N-glycan modification in MATP-KD cells compared to vehicle or scrambled siRNA-treated cells before and after the EndoH treatment (S4 Fig), suggesting that another mechanism is involved in the reduction of tyrosinase activity in MATP-KD cells. We used electron microscopy to detect changes in the intracellular vesicles, especially the melanosomes. Compared to scrambled siRNA-treated control cells, in which the melanized melanosomes (stage III or IV) were frequently observed and there were few immature melanosomes (Fig 3D, scrambled siRNA, white arrowheads vs. black arrowheads), the cells treated with MATP siRNA displayed mostly immature, poorly melanized melanosomes (Fig 3D, MATP siRNA, black arrowheads) without any morphological changes. These results indicate that MATP in the melanosomes is closely related to melanin production, and thus, the loss of MATP increases the poorly pigmented early melanosomes.

### MATP regulates melanosomal pH

**Fig 5:**
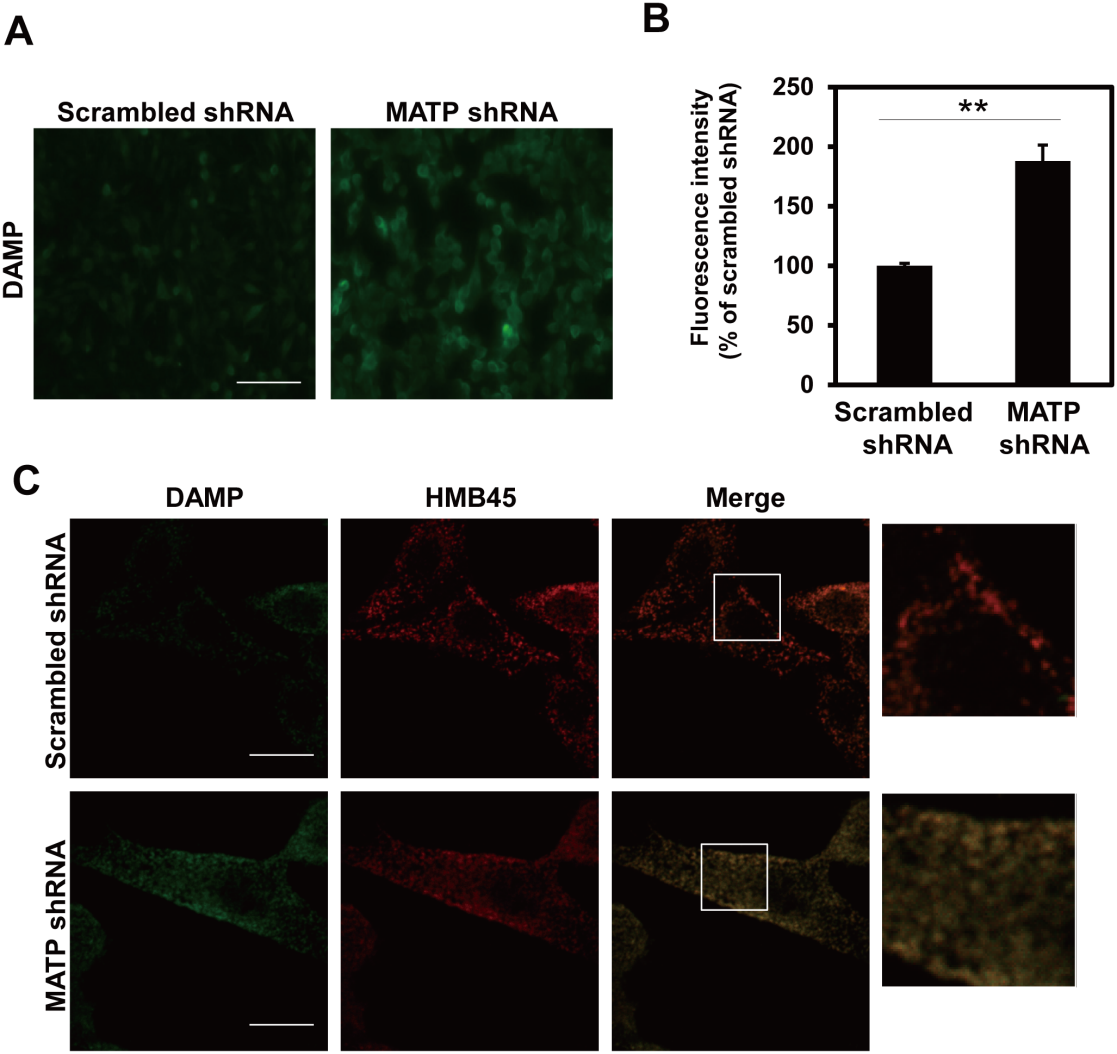
The knockdown of MATP acidifies the melanosomal pH. (A) MNT-1 cells stably expressing scrambled or MATP shRNAs were treated with the pH indicator DAMP for 30 min. An anti-DNP antibody was used to detect DAMP by fluorescence microscopy. Scale bars = 200 μm. (B) The fluorescence intensity was calculated with ImageJ software (http://rsbweb.nih.gov/ij/download.html). The data are representative of three independent experiments (*, *P* < 0.05). (C) MNT-1 cells stably expressing scrambled or MATP shRNAs were co-stained with anti-DNP and anti-HMB45 antibodies after DAMP treatment for 30 min and then visualized by confocal microscopy. Each inset was magnified. Scale bars = 10 μm.

Considering that MATP is predicted to be a putative sugar transporter that utilizes the proton gradient, and melanosomal pH can be directly affected by changes in the proton gradient, we examined whether melanosomal pH changed after treatment with MATP shRNA. We measured the melanosomal pH using a DAMP assay in MNT-1 cells stably expressing scrambled or MATP shRNAs. In the MATP-KD cells, the DAMP signal, which indicates low pH, was much stronger than that in the scrambled control cells (Fig 5A and 5B). We co-stained the cells with anti-DNP and anti-HMB45 antibodies for DAMP and melanosomes, respectively, and found that both signals were well merged (Fig 5C). These data suggest that MATP-KD acidifies the melanosomal pH, which is associated with lowered tyrosinase activity. Tyrosinase is a representative metalloprotein requiring a copper ion as a cofactor; metal coordination into proteins is sensitive to pH. We hypothesize that the acidification of melanosomal pH by MATP-KD could inhibit the incorporation of copper into tyrosinase and thus reduce tyrosinase activity. To test this hypothesis, we knocked down the endogenous MATP using siRNA in MNT-1 cells and performed the *in vitro* L-DOPA oxidase activity assay of tyrosinase using cell lysates with or without copper sulfate. The copper treatment completely recovered the tyrosinase activity that was significantly reduced in MATP-KD cells (Fig 4), suggesting that the reduced tyrosinase activity in MATP-KD cells is due to improper copper binding to tyrosinase under the acidic conditions in melanosomes and that the acidic conditions are due to MATP deletion.

## Discussion

**Fig 6:**
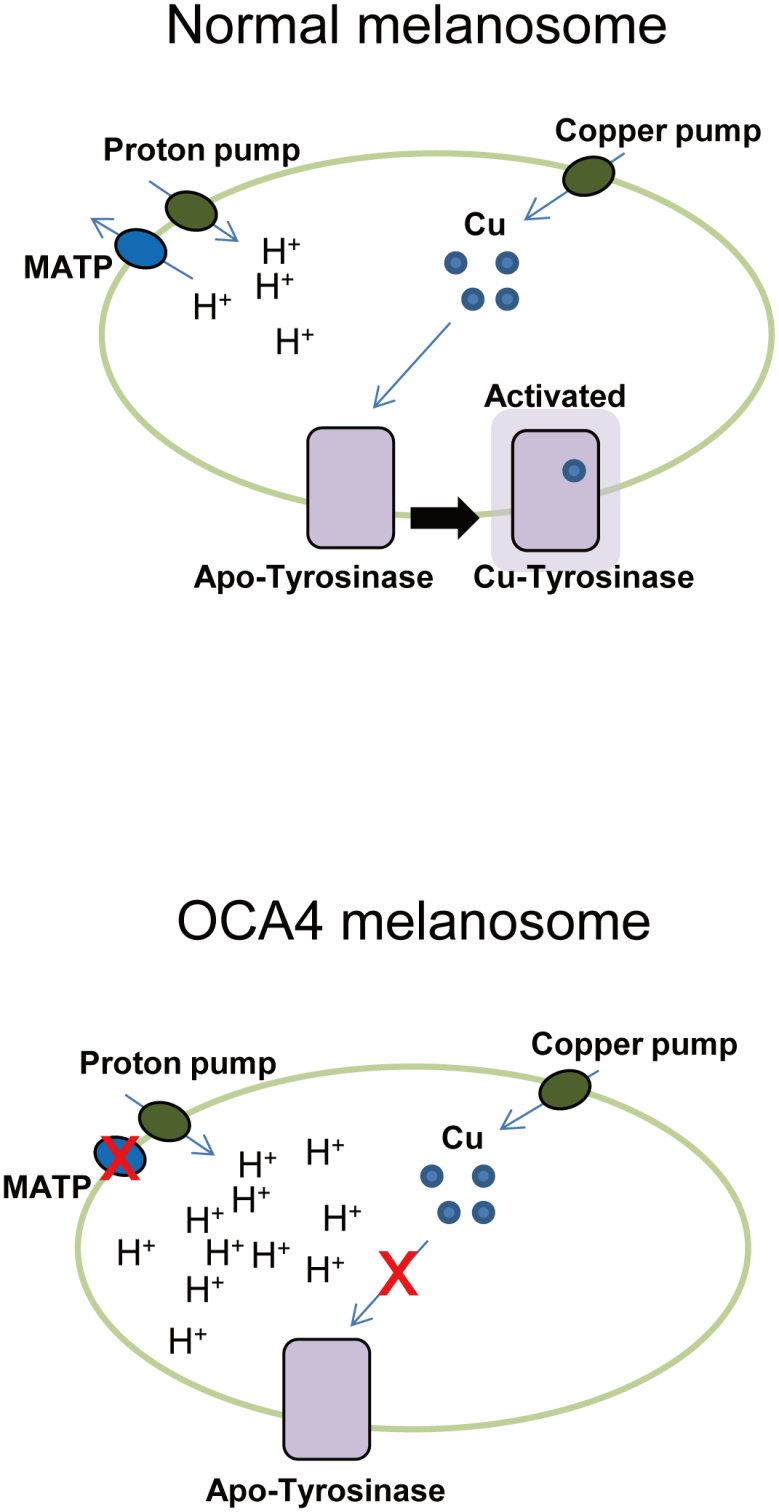
Schematic models of MATP function as a putative transporter in regulating melanosomal pH in normal and OCA4 conditions. Under normal conditions, MATP elevates the melanosomal pH by functioning as a transporter using a proton gradient. Under this condition, copper can bind to tyrosinase (Cu-Tyrosinase), resulting in active tyrosinase. In OCA4 melanosomes, MATP does not function properly and the melanosomal lumen becomes acidic. Under this condition, copper cannot incorporate into tyrosinase and tyrosinase activity is reduced. Apo-Tyrosinase, tyrosinase without copper; Cu-Tyrosinase, tyrosinase with copper.

We investigated the role of the MATP protein in melanogenesis. Among the *SLC45A* gene families, only the *SLC45A2* gene, encoding the MATP protein, is enriched in melanotic cells. In MATP-KD generated by siRNA, melanin production was down-regulated and the number of poorly pigmented melanosomes increased, without significant alterations in the expression of the melanogenesis-related genes and the morphology of the melanosomes. Simultaneously, the MATP-KD was associated with reduced tyrosinase activity. A DAMP assay demonstrated that MATP-KD lowered the melanosomal pH, which may affect tyrosinase activity. Tyrosinase activity was readily recovered by copper treatment, suggesting that MATP can have an effect on tyrosinase activity by maintaining the proper melanosomal pH for copper to bind to tyrosinase. Finally, we proposed a model explaining how MATP acts as a transporter to regulate melanosomal pH and thus tyrosinase activity (Fig 6).

A previous study using melanocytes derived from *uw*/*uw* mice revealed that the melanocytes 1) had an abnormally large and more dendritic morphology, 2) continuously secreted dark vesicles containing tyrosinase, TYRP-1 and TYRP-2, 3) possessed tyrosinases with only 20% of the activity of tyrosinases from wild type melanocytes, and 4) had swollen and poorly melanized melanosomes [22]. Here, we found that MATP-KD reduced tyrosinase activity and induced poorly melanized melanosomes, which is consistent with the results shown for the *uw*-mutant melanocytes. However, in our experiments, no significant morphological changes or secreted dark vesicles were found (Fig 3A). In addition, swollen melanosomes were not detected by electron microscopy (Fig 3D). These findings may result from cell type differences (mouse vs. human) and/or incomplete knockdown efficiency. Human melanocytes are distinct from mouse melanocytes. Briefly, mouse melanocytes are located in the hair bulb or bulge, but human melanocytes exist in the basal layer of the skin epidermis, including the hair bulb or bulge [24, 25]. Identical stimuli sometimes lead to different cellular responses depending on the cell type [24, 25]. In this study, we used a siRNA system to knockdown MATP. Although MATP siRNA was transfected and efficiently knocked down the endogenous *SLC45A2* transcript, thereby reducing the protein level, approximately half of the *SLC45A2* transcript was still expressed (S3 Fig), which might result in partial phenotypic effects.

The melanosomal pH is regulated by “pumps” and “transporters” [26]. Pumps use ATP to move substrates to an opposite substrate gradient across a membrane. The V-ATPases that mediate proton influx across the membrane to regulate melanosomal pH have been previously reported [26]. Transporters facilitate substrate movement across the membrane via two energy sources: one is a substrate gradient and the other is a proton gradient generated by the V-ATPases [27, 28]. Given that MATP has a sucrose transporter motif, but not an ATP biding motif, it was assumed that MATP is a transporter and not a pump [16]. As a transporter, MATP seems to utilize the proton gradient to transport its putative sugar-like substrate as demonstrated in the result that MATP-KD led to an acidic melanosomal pH (Fig 5) and as suggested in our model (Fig 6). This result is consistent with a recent report, which claims that MATP transports sucrose via a proton-coupled transport mechanism when heterologously expressed in *Saccharomyces cerevisiae* [29]. The sugar substrate of MATP may be supplied by reversible reactions of glycosylation on many vesicular and secretory proteins because the serial attachment and detachment of sugars is an essential step of protein glycosylation and many disaccharides are generated in this process [30]. Therefore, it is possible that as a proton/sugar symporter, MATP transports these sugars from the melanosome to the cytoplasm using a proton gradient generated by a proton pump. This putative MATP transporting mechanism is similar to that of the plant sucrose/proton symporters, with which MATP shares a high amino-acid identity [16,17,19]. However, other groups proposed a model in which MATP imports sucrose into the melanosome to reduce the hypotonic stress induced by tyrosine polymerization to melanin [18, 29]. There is no doubt that the osmotic status within the melanosome is changed during melanization, and several melanosomal pumps or transporters, including MATP and other cation/proton antiporters (for example, sodium/hydrogen exchangers) [31], may work together to regulate melanosomal pH and to maintain homeostasis within the melanosome for a step-wise melanogenesis. siRNA treatment targeting the potassium-dependent sodium calcium exchanger reduces melanin production [32]. Together with our results, both sugar and cation appear to be crucial for melanogenesis, and it may be possible that the expression levels and patterns of the transporters are distinct dependently on the stage of melanosome maturation. Further studies, such as chasing the osmolytes within the melanosome before and after MATP-KD, will provide more precise information regarding the involvement of MATP in substrate transport and thus in melanogenesis. In addition, under an electron microscope, we observed that the melanosomes in MATP-KD cells were mostly stalled at stage II. It would also be interesting to test if MATP can directly regulate the maturation process of melanosomes by controlling the sugar-induced melanosomal osmotic potential as suggested previously [33, 34].

Copper ions are imported into the cytoplasm via the copper transporters, which belong to the SLC31 family on the plasma membrane; are immediately bound by intracellular chaperones; and are then transferred to other molecules, such as ATP7A on melanosomes, to activate tyrosinase [35, 36]. Copper ions are present as Cu(I) in those steps because endogenous reductases and components residing on the cell surface reduce copper ions prior to their transportation into the cytoplasm, and the cytoplasm itself prefers the reducing condition [35]. Unlike the cytoplasm, we speculate that within the melanosome, Cu(II) may be dominant compared with Cu(I) because the melanosomal lumen is oxidative to support melanin production [37]; therefore Cu(II) could be present in the active center of tyrosinase. To activate tyrosinase in this condition, Cu(II) should be reduced to Cu(I), and the initial intermediate L-DOPA may play a role as a reductor, resulting in the switch from Cu(II) to Cu(I) within the active site of tyrosinase. We showed that MATP-KD lowered the melanosomal pH (Fig 5). In this condition, tyrosinase activity was decreased (Fig 4, left panel), and the decreased tyrosinase activity was recovered when copper was exogenously introduced into the cell lysates (Fig 4, right panel). Because the acidic pH could alter the charge status of the amino acids, for example, histidine in the active site of tyrosinase, which in turn could alter the incorporation of the metal ion into that site and the subsequent tyrosinase activity, we assume that copper (perhaps Cu(II)) was not properly incorporated into tyrosinase within the melanosomes in MATP-KD cells. For the *in vitro* L-DOPA oxidase activity assay, we lysed the MATP-KD cells using a neutral pH lysis solution in which cytosolic copper ions could bind to many other cellular proteins, including tyrosinase, causing the depletion of available copper ions for tyrosinase. We supplied CuSO4 in the *in vitro* L-DOPA oxidase activity assay to mimic the melanosome luminal condition, where Cu(II) may be preferentially present. Once the pH is neutral, the incorporation of Cu(II) into the tyrosinase active site is normal, and the switch from Cu(II) to Cu(I) in the active center of tyrosinase can be mediated by exogenously added L-DOPA, as proposed in normal melanosomes. Some portion of Cu(II) might be reduced to Cu(I) before being incorporated into apo-tyrosinase in the *in vitro* assay system for two reasons: 1) a sufficient amount of L-DOPA was supplied as a substrate; 2) the cell lysates used in this assay might contain proteins and cellular organic compounds with reducing potential, such as glutathione. Although we speculate that Cu(II) may be dominant compared with Cu(I) within normal melanosomes and the low melanosomal pH in the MATP-KD cells could affect the incorporation of copper ions, it is difficult to precisely measure the portion of Cu(II)/Cu(I) and thus definitively describe the status of copper ions in normal or OCA4 melanosomes. Therefore, Cu, rather than CuI(I) or CuII(II), is a better description in this model (Fig 6).

The pH is a well-known factor that is crucial for melanin synthesis in many aspects. In early stage melanosomes, the acidic pH contributes to stabilize L-DOPA by preventing auto-oxidation and the elevating pH according to melanosome maturation optimizes the tyrosinase activity for melanin production [37–39]. Considering that an acidic melanosomal pH reduces tyrosinase activity and prevents L-DOPA oxidation [26, 39], the MATP-KD could influence on melanogenesis not only by inhibiting tyrosinase activity via altering copper incorporation but also by changing the portion of L-DOPA. L-DOPA, together with L-tyrosine, plays roles as a substrate for melanogenesis and as a hormone-like activator of the process [40, 41]. The altered L-DOPA level by MATP-KD may also affect on the endogenous level of melanogenesis. In different culture media, different concentrations of L-tyrosine are included. As L-tyrosine and L-DOPA are activators of melanogenesis, the different levels of L-tyrosine due to different media or stimuli used may also affect the endogenous level of melanogenesis and thus the effective level of MATP-KD. It may be worth testing the effect of MATP-KD in other melanoma cell lines or primary melanocytes, which are cultured under different medium conditions, to test this possibility.

In conclusion, we showed that MATP is highly expressed in melanocytic lineages, is located in the melanosome, and is involved in maintaining the proper melanosomal pH to allow the correct incorporation of copper ions into the tyrosinase active site. These findings reveal a mechanism explaining how the MATP protein plays a role in melanin production and, by extension, how its mutation is associated with OCA4, providing new insights for therapeutic approaches to OCA4.

## Materials and Methods

### Cell culture and materials

The human melanoma MNT-1 cell line was kindly provided by Dr. Ai-Young Lee at Dongguk University, who originally received it as a gift from Dr. Vincent J. Hearing at National Institutes of Health, Bethesda, Maryland, USA [42]. The cells were cultured at 37°C in MEM (Gibco, Carlsbad, CA, USA) containing 20% FBS, 10% DMEM (Lonza, Basel, Switzerland), 20 mM HEPES and antibiotics. The WM266-4 human melanoma cells were obtained from the American Type Culture Collection (ATCC, Manassas, VA, USA). The SK-MEL-1, SK-MEL-2, SK-MEL-3, and SK-MEL-5 human melanoma cell lines were obtained from the Korean Cell Line Bank (KCLB, Seoul, Korea). Those cells were maintained at 37°C in RPMI1640 medium (Gibco) containing 10% FBS and antibiotics. Normal human melanocytes (Cascade Biologics, Portland, OR, USA) were cultured in M254 medium (Cascade) containing human melanocyte growth supplement-2 (Cascade). Endo H and PNGase F were purchased from New England Biolabs (Hitchin, UK).

### Determination of melanin levels and the L-DOPA oxidase activity assay of tyrosinase

Cell pellets were dissolved in 1 N NaOH for 1 hour at 80°C. The melanin levels were determined by measuring the absorbance at 450 nm. For the L-DOPA oxidase activity assay of tyrosinase, the cells were harvested with 1% NP-40 in PBS and clarified by centrifugation for 20 minutes at 16,000 ×*g*. The supernatants were incubated with 2 mg/ml L-DOPA for 1 hour, and the absorbance at 450 nm was measured.

### Western blot analysis and fluorescence microscopy

Cells were lysed in a buffer containing 0.05 M Tris-HCl (pH 7.5), 0.15 M NaCl, 0.01 M MgCl_2_, 1% NP-40 and a protease inhibitor cocktail (Sigma, St. Louis, MO, USA) and were subjected to centrifugation. The protein content was quantified using the BCA assay. An aliquot of 10–20 μg of protein was loaded into each well of an SDS-PAGE gel. For immunoblotting, the SDS-PAGE gel was electroblotted to a PVDF membrane. The following antibodies were used: anti-MATP (Abnova, Taipei, Taiwan); anti-MATP F-4 (Santa Cruz Biotechnology, Santa Cruz, CA, USA); anti-MATP H-130 (Santa Cruz Biotechnology); anti-EEA1 (Abcam, Cambridge, UK); anti-TYRP-1, anti-LAMP1, anti-TNG46, anti-TYRP-2, and anti-GAPDH (Santa Cruz Biotechnology); anti-Tyr (Upstate Biotechnology, Lake Placid, NY, USA); and anti-TA99; anti-HMB45 (GenTex, Irvine, CA, USA). The MATP recombinant protein (Abnova) was purchased and used for pre-absorption of anti-MATP antibody (Abnova). For fluorescence microscopy, cells were cultured on Lab-Tek chamber slides (Nunc, Waltham, MA, USA) and then fixed with 4% paraformaldehyde in PBS, permeabilized with 0.1% Triton X-100 in PBS containing 1% BSA for 5 minutes, and incubated with each antibody. Fluorescence was detected after secondary staining with the Alexa Fluor 488-conjugated F(ab')2 fragment of goat anti-mouse IgG (Invitrogen) and the Alexa Fluor 594-conjugated F(ab')2 fragment of goat anti-rabbit IgG (Invitrogen).

### siRNA and shRNA transfection

MNT-1 cells were transiently transfected with 100 nM ON-TARGETplus SMARTpool siRNA (Thermo Fisher Scientific, Waltham, MA, USA) using Lipofectamine RNAimax (Invitrogen) according to the manufacturer’s instructions. For the shRNA-expressing stable cell line, a MATP shRNA plasmid was constructed using the MATP sequence (5’-GUACGAGUAUGGUUCUAUC-3’). After transfection with this plasmid, selection was performed for 2 weeks with 200–1000 μg/ml zeocin (Genolution, Seoul, Korea) according to the manufacturer’s instructions.

### Quantitative real-time PCR (RT-qPCR)

Total RNA (1-2-μg) was reverse-transcribed into cDNA using ReverTra Ace (Toyobo, Osaka, Japan) and an oligo(dT) primer. The gene expression analysis was performed using TaqMan Universal Master Mix and TaqMan Gene Expression Assays (Applied Biosystems, Foster City, CA, USA). The primers are listed in S1 Table.

### Transmission electron microscopy

Confluent flasks of MNT-1 cells were fixed using Karnovsky fixative (Electron Microscopy Sciences, Hatfield, PA, USA) for 30 minutes at RT, harvested by centrifugation and then embedded in a low-melting-point agarose matrix (Sigma). After washing with PBS, the agarose-embedded cells were post-fixed in 2% osmium tetroxide and stained with uranyl acetate. Then, the specimens were dehydrated through a graded ethanol series and embedded in Embed-812 (Electron Microscopy Sciences) at 60°C for 72 hours. Ultrathin sections (approximately 70–90 nm) were stained using uranyl acetate and lead citrate and then observed using a transmission electron microscope (TEM), JEM-1200 EX (Joel, Tokyo, Japan).

### DAMP assay

The Acidic Granule kit was purchased from Oxford Biomedical Research (Oxford, MI, USA). Cells were incubated with 30 μM DAMP [N-(3-((2,4-dinitrophenyl)amino)propyl)-N-(3-aminopropyl)methylamine], Oxford Biomedical Research) for 30 minutes, fixed with 3% (wt/vol) paraformaldehyde and washed with 50 mM ammonium chloride. After permeabilization with 0.1% Triton X-100 in PBS, an anti-DNP antibody (Oxford Biomedical Research) was added according to the manufacturer’s instructions.

### Statistical analysis

A two-tailed Student’s t-test was used to analyze the differences between two groups.

## Supporting Information

S1 Fig The expression of MATP protein in melanotic cells. The expression of MATP protein in primary melanocytes and in various melanoma cell lines was analyzed by western blotting using two anti-MATP antibodies, F-4 (upper panel) and H-130 (lower panel).
(TIF)

S2 Fig The MATP protein is located in the melanosomes. MNT-1 cells were stained with anti-HMB45 or anti-TA99 antibodies together with an anti-MATP (H-130) antibody. The inset is magnified. Scale bars = 10 μm.
(TIF)

S3 Fig The effect of MATP-KD on the expression of melanogenesis-related genes. The mRNA expression levels of melanogenesis-related genes in MNT-1 cells were analyzed by RT-qPCR at day 4 post-treatment of MATP siRNA. The data are representative of three independent experiments (***, *P* < 0.005). TYR, tyrosinase; TYRP-1, tyrosinase related protein-1; PMEL17, premelanosome protein 17; MITF, microphthalmia-associated transcription factor.
(TIF)

S4 Fig Deglycosylation assay of tyrosinase. The lysates of MNT-1 cells treated with scrambled or MATP siRNAs were incubated with or without Endo H for 24 hours, and tyrosinase protein was detected using an anti-TYR antibody.
(TIF)

S1 Table Primer list used for quantitative real-time PCR analysis. (XLSX)
