# Supplementary figures and images for: Membrane-Associated Transporter Protein (MATP) Regulates Melanosomal pH and Influences Tyrosinase Activity
Source: PLoS One. 2015 Jun 9;10(6):e0129273. doi: 10.1371/journal.pone.0129273 (PMC4461305; doi:10.1371/journal.pone.0129273)

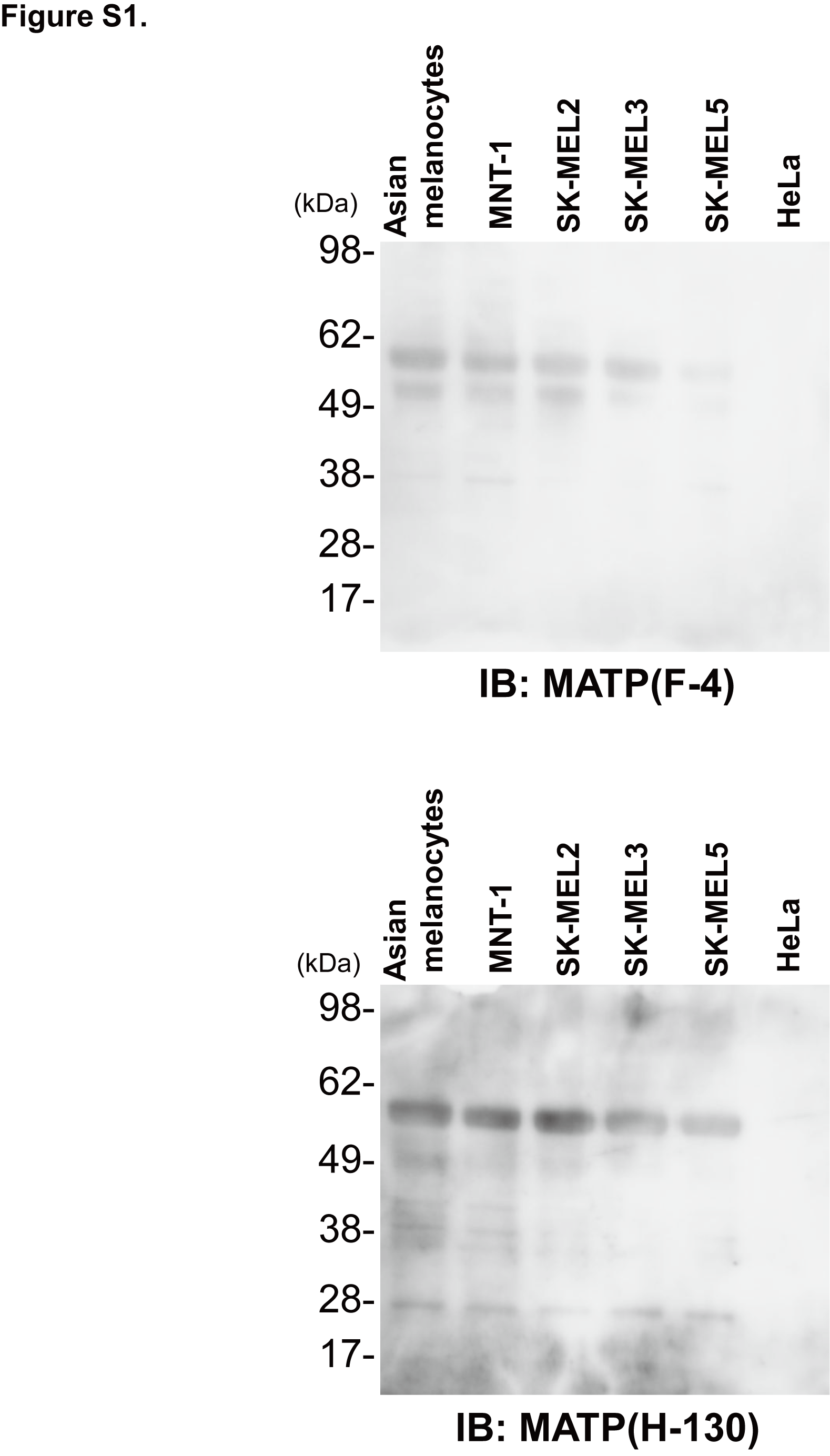

Supplement: S1 Fig — The expression of MATP protein in primary melanocytes and in various melanoma cell lines was analyzed by western blotting using two anti-MATP antibodies, F-4 (upper panel) and H-130 (lower panel). (TIF) [file pone.0129273.s001.tif]

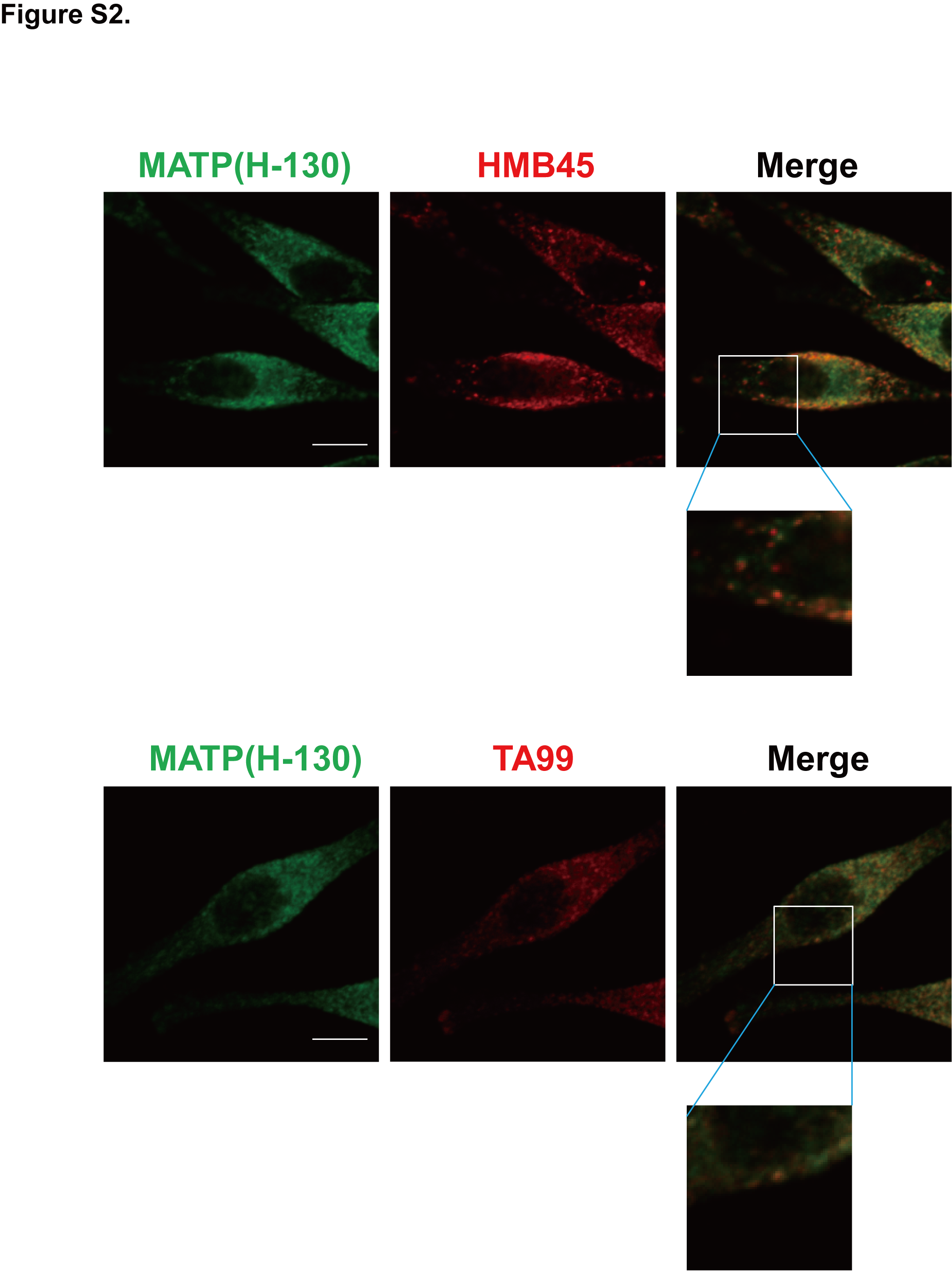

Supplement: S2 Fig — MNT-1 cells were stained with anti-HMB45 or anti-TA99 antibodies together with an anti-MATP (H-130) antibody. The inset is magnified. Scale bars = 10 μm. (TIF) [file pone.0129273.s002.tif]

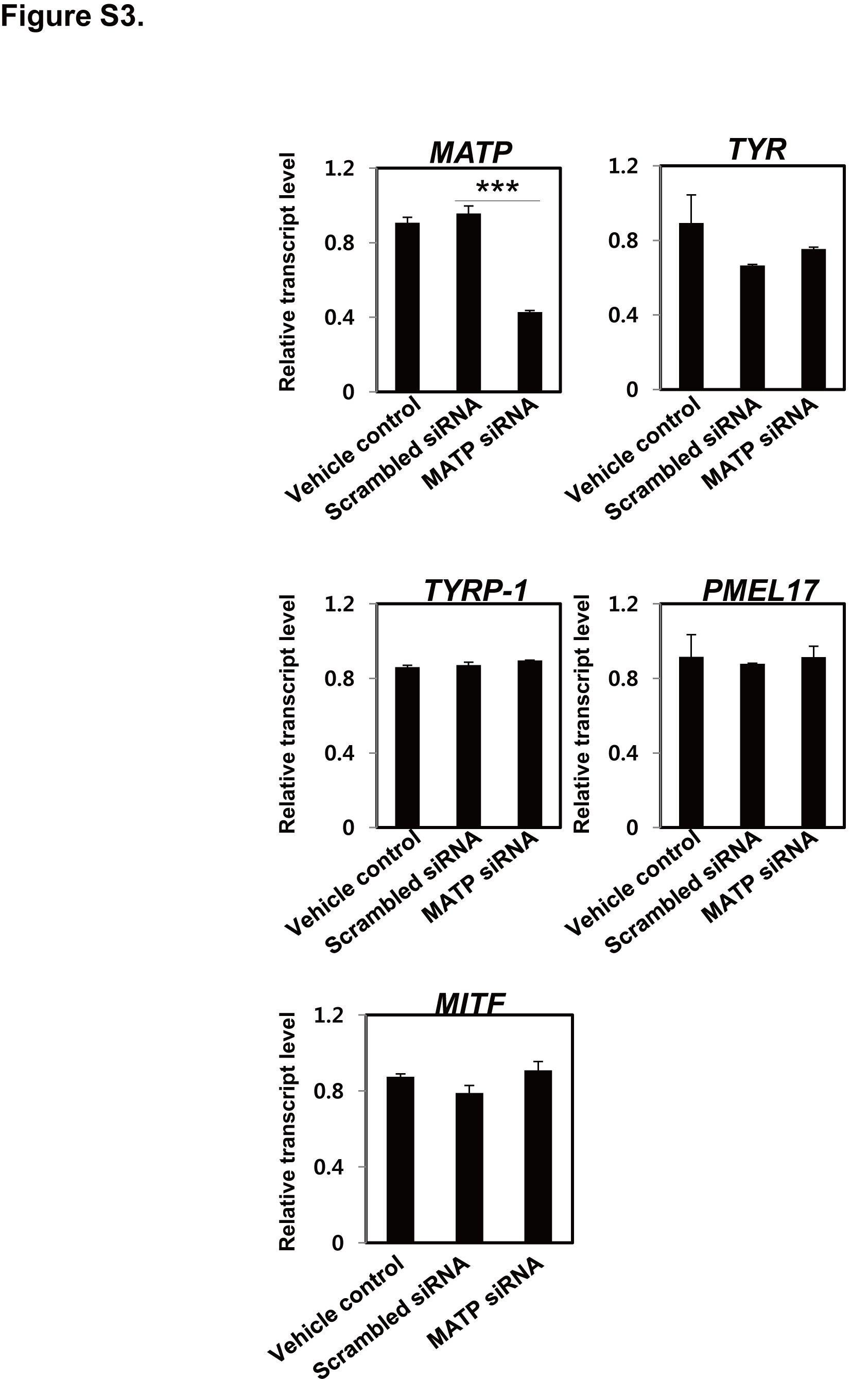

Supplement: S3 Fig — The mRNA expression levels of melanogenesis-related genes in MNT-1 cells were analyzed by RT-qPCR at day 4 post-treatment of MATP siRNA. The data are representative of three independent experiments (***, P < 0.005). TYR, tyrosinase; TYRP-1, tyrosinase related protein-1; PMEL17, premelanosome protein 17; MITF, microphthalmia-associated transcription factor. (TIF) [file pone.0129273.s003.tif]

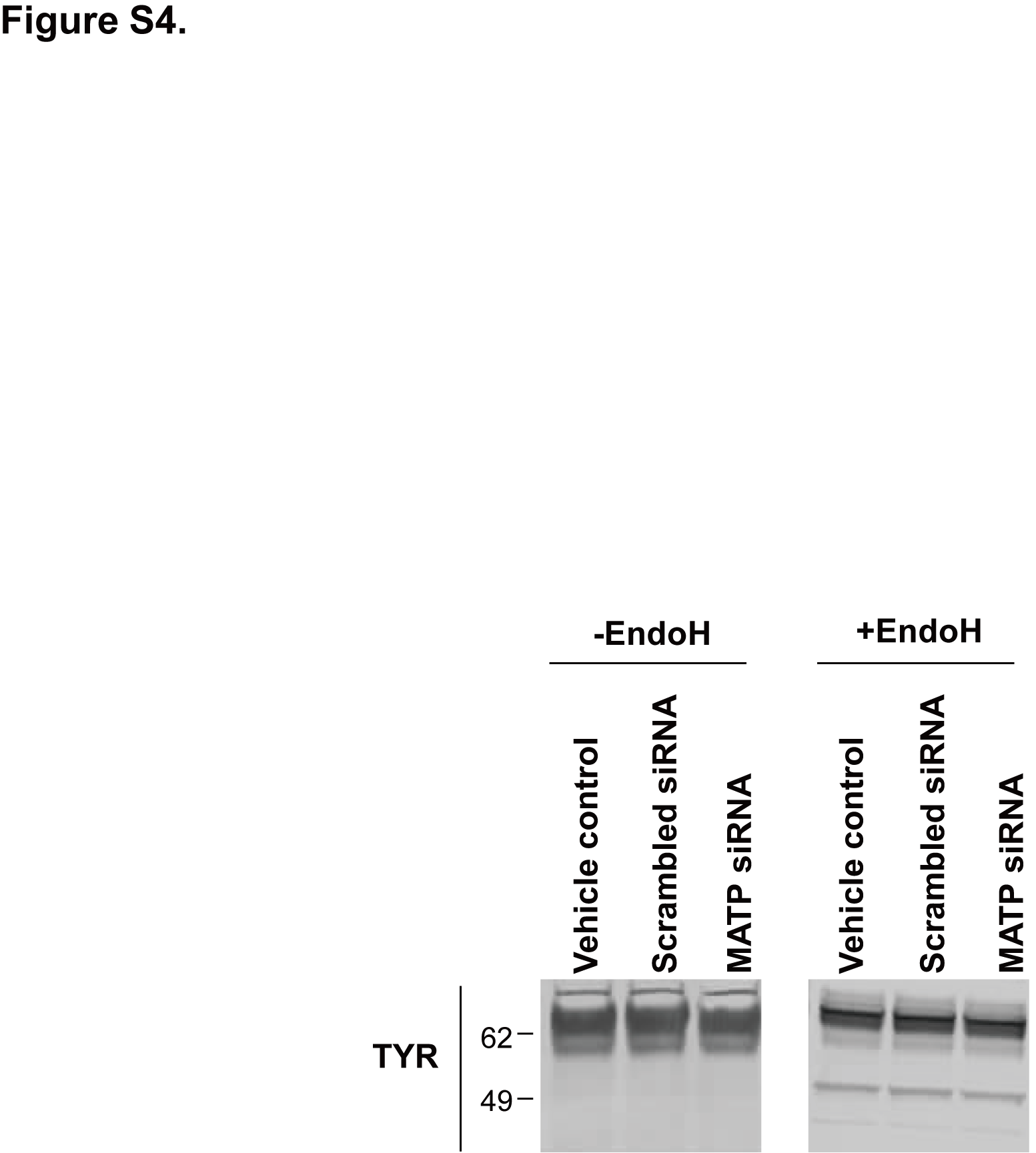

Supplement: S4 Fig — The lysates of MNT-1 cells treated with scrambled or MATP siRNAs were incubated with or without Endo H for 24 hours, and tyrosinase protein was detected using an anti-TYR antibody. (TIF) [file pone.0129273.s004.tif]
